# Comparison of oral human papilloma virus detection methods among Hispanic adults

**DOI:** 10.1002/cre2.522

**Published:** 2021-12-29

**Authors:** Maira A. Castañeda‐Avila, Cynthia M. Pérez, José A. Vivaldi‐Oliver, Elba C. Díaz‐Toro, Ana Patricia Ortiz

**Affiliations:** ^1^ Department of Population and Quantitative Health Sciences University of Massachusetts Chan Medical School Worcester Massachusetts USA; ^2^ Department of Biostatistics and Epidemiology Graduate School of Public Health, Medical Sciences Campus, University of Puerto Rico San Juan Puerto Rico; ^3^ Department of Restorative Sciences School of Dental Medicine, Medical Sciences Campus, University of Puerto Rico San Juan Puerto Rico; ^4^ Division of Cancer Control and Population Sciences University of Puerto Rico Comprehensive Cancer Center San Juan Puerto Rico

**Keywords:** HPV collection method, oral cytobrush, oral rinse, periodontitis

## Abstract

**Objectives:**

Oral human papilloma virus (HPV) infection is associated with nearly three‐quarters of all oropharyngeal cancers in the United States. Research also suggests its association with periodontal disease. There are limited studies evaluating differences in HPV detection methods; however, oral rinse is considered the most sensitive detection method. We compared HPV detection by self‐collected oral rinse versus self‐collected cytobrush and assessed whether the strength of association between periodontitis and HPV is modified by the collection method.

**Materials and Methods:**

Data from a cross‐sectional study of Hispanic adults in Puerto Rico (*n* = 346) who provided oral rinse and cytobrush samples for oral HPV detection and were clinically evaluated for periodontitis. The agreement between the oral mouthwash and cytobrush methods was assessed using the Kappa (*κ*) statistic. Logistic regression models were used to determine if the association between HPV infection and other risk factors varied by oral sample collection method.

**Results:**

HPV prevalence was slightly higher using cytobrush than oral rinse (5.8% vs. 4.3%). The sensitivity of cytobrush to detect oral HPV was 64.7%, and the specificity was 97.4%. We observed a *κ* of 0.61 (95% confidence interval [CI]: 0.45–0.78), indicative of fair to good agreement between the two collection methods. The association between oral HPV infection and periodontitis severity was stronger when using the oral rinse collection method (odds ratio [OR] = 3.23, 95% CI: 1.06–9.84); the association was not statistically significant for cytobrush (OR = 1.96, 95% CI: 0.68–5.65).

**Conclusions:**

These findings support the significance of choosing the most suitable collection method in oral HPV‐related studies. Selecting the most appropriate collection method is an essential criterion in oral HPV‐related studies.

## 1 INTRODUCTION

A vast majority of oropharyngeal cancer cases in the United States are associated with oral Human Papilloma Virus (HPV) infection (Bouvard et al., 2009; Pytynia et al., 2014; Senkomago et al., 2019). The rising rates of HPV‐associated oropharyngeal cancers are a global public health concern despite the introduction of an effective HPV vaccine (Chaturvedi et al., 2011; You et al., 2019). Previous studies have compared HPV detection rates using different DNA extraction kits and amplification methods, finding nested PCR is the most appropriate method for detecting HPV DNA (Božić et al., 2020). In addition to adequate detection and extraction methods, selecting an acceptable sampling method is essential. There are various sampling methods for the detection of oral HPV, including oral rinse, nylon swab, biopsy specimen, cytobrush, and oral mucosal scraping; however, it is unclear which is the best oral HPV detection method (Chikandiwa et al., 2018; Combes et al., 2017; de Souza et al., 2018; Donà et al., 2019; Ong et al., 2014; Steinau et al., 2012). Variability in the rates of oral HPV detection through these collection methods may affect the results of HPV‐related studies. Despite there is no universally agreed‐upon method, oral rinse is considered the most suitable sample method since it provides the highest HPV detection rate in epidemiologic studies (Rosenthal et al., 2017). A previous study among 163 men who have sex with men found that the agreement for HPV status between oral rinse and brushing is poor (Donà et al., 2019).

Beyond its association with oropharyngeal cancers, HPV infection has also been associated with periodontal disease (Ortiz et al., 2018; Wiener et al., 2015). This chronic oral condition affects approximately 47% (~65 million) of adults in the United States (Eke et al., 2012). Nonetheless, epidemiologic studies are sparse and have yielded inconsistent results (Ortiz et al., 2018, 2019; Wiener et al., 2015). A recent review article highlights the contradictory evidence regarding the association between periodontitis and oral HPV infection. These potentially conflictive findings are attributed to the diversity of methodologies to determine periodontitis and oral HPV infection and the consideration of periodontitis severity (Ortiz et al., 2019). The biological plausibility for this association includes the possibility of periodontal tissue as a potential reservoir for oral HPV infection and facilitation of HPV persistence by chronic inflammation of the tissue (Ortiz et al., 2019; Syrjänen, 2018). A recent meta‐analysis evaluating the association between HPV and periodontitis found a strong association (odds ratio [OR] = 3.65, 95% confidence interval [CI] = 1.67–8.01) between any oral HPV infection and periodontitis, although an inconclusive positive association between periodontitis and oral high‐risk HPV infection (Ali et al., 2020). The aims of this study were: (1) to compare the detection of HPV by oral rinse and self‐collected cytobrush and (2) to determine whether the strength of association between HPV and periodontitis is modified by the oral sample collection method in a sample of Hispanic adults in Puerto Rico.

## 2 MATERIALS AND METHODS

### 2.1 Study design and study subjects

A subset of 346 consecutive Hispanic participants recruited within a cross‐sectional study performed in Puerto Rico between 2014 and 2016 who provided oral rinse and cytobrush samples of the gums comprised the study population of interest (Ortiz et al., 2018). The parent cross‐sectional study was designed to determine the prevalence of oral HPV infection in the San Juan Overweight Adults Longitudinal Study (SOALS) (Ortiz et al., 2018). SOALS is a prospective cohort study designed to evaluate the bidirectional association between the progression of periodontitis and glucose abnormalities among overweight and obese adults aged 40–65 years (Andriankaja et al., 2015). The Institutional Review Board of the University of Puerto Rico Medical Sciences Campus approved the study, and all adults provided written consent before participation in the study.

### 2.2 Data collection procedures

Participants were asked to complete a self‐administered questionnaire consisting of 10 questions regarding their current oral hygiene practices: time since last tooth brushing, type of toothbrush and toothpaste used, and mouthwash use. In addition, participants completed a computer‐assisted self‐interview to gather information on oral HPV infection risk factors, sexual behavior, and drug use.

#### 2.2.1 Oral samples for HPV determination

Self‐collected oral mouthwash samples were collected using the NHANES methodology (Gillison et al., 2012). A 50‐ml sterile collection cup filled with 10 ml of Scope (original mint flavor) was provided to each participant. They were asked to rinse/gargle with the mouthwash for 30 seconds, spit the mouthwash into the collection cup while trying not to spill the liquid, and close the cup tightly. Afterward, participants used a cytobrush (QIAGEN) to self‐collect an oral sample (Read et al., 2^012^). They gently brushed the gums' inner and outer areas for at least 10 strokes. Samples were obtained using the digeneHc2 DNA collection device from QIAGEN. Both samples were performed during the visit and supervised by the study coordinators.

#### 2.2.2 HPV typing

HPV typing was performed at the University of California at San Francisco HPV Virology Core Laboratory using polymerase chain reaction (PCR) by dot‐blot hybridization with modified L1 consensus primers (MY09/MY11) that identify 38 different type‐specific probes, including oncogenic and non‐oncogenic HPV types (Bouvard et al., 2009; Ortiz et al., 2018). After thawing, the samples were processed for DNA using the Gentra Pure gene Buccal Cell Kit (QIAGEN) following the manufacturer's instructions. The quality of the DNA was evaluated through β‐globin gene amplification. A sample was considered unsatisfactory if it was negative for β‐globin. From this analysis, only five samples, using the oral rinse method, were unsatisfactory. A sample was considered HPV positive if it was positive for the consensus probes or any specific HPV type probe. Amplification of a solution containing all the above components, except for sample DNA, or DNA from cell lines with and without HPV, was used as control.

#### 2.2.3 Periodontal assessment

Periodontitis was evaluated by clinical measurements. These measurements include clinical attachment loss (CAL) and probing depth (PD) at six sites for all teeth, excluding the third molars (Gillison et al., 2012). Periodontitis was defined using the Centers for Disease Control/American Academy of Periodontology definition (Borrell & Talih, 2012; Palefsky et al., 2001). Severe periodontitis was defined as ≥2 interproximal sites with CAL ≥6 mm (not on the same tooth) and ≥1 interproximal site with PD ≥5 mm. Full mouth clinical exams were conducted by a dental examiner previously calibrated by the NHANES reference examiner (The Centers for Disease Control and Prevention [CDC, 2018]).

### 2.3 Statistical analysis

Sensitivity and specificity were estimated using 95% binomial confidence intervals (CIs). Receiver operating characteristic (ROC) analysis was used to determine the area under the curve. The Kappa (*κ)* statistic quantified the level of agreement between the oral mouthwash and cytobrush methods. Logistic regression models were used to assess if the association between HPV infection and severe periodontitis varied by oral sample collection method. The models were adjusted by age, gender, time since last tooth brushing, and number of sexual partners as they were significantly associated (*p* < .05) in the bivariate analysis to oral HPV infection and periodontitis. The addition of other potential confounders did not change the effect estimates.

## 3 RESULTS

### 3.1 Characteristics of the study population

The mean age of participants was 53.6 ± 6.7 years; 73% were women, and 49% were married or living with someone. Nearly 15% of participants were current smokers and binge drinkers. Approximately 1 in 5 participants had at least 10 lifetime sexual partners, while the vast majority reported less than 2 lifetime oral sex partners. Nearly 20% of participants had severe periodontitis. On the interview day, less than two‐thirds of participants brushed their teeth within 5 or more hours before the sample collection, and nearly half used mouthwash before study participation (Table 1).

**Table 1:** Demographic, lifestyles, and clinical characteristics of the study population (*n* = 346)

Characteristics	*n* (%)
Women	254 (73.4)
Age (years)	
40–49	107 (31.0)
50–64	250 (69.0)
Marital status	
Single	55 (15.9)
Married/cohabiting	168 (48.6)
Divorce/widowed	123 (35.6)
≤12 years of education	190 (54.9)
Annual family income	
<$20,000	177 (51.2)
≥$20,000	169 (48.8)
Current smoking	60 (17.4)
Current drinking	152 (43.9)
Binge drinking	50 (14.5)
Marijuana use in the last 30 days (≥2 times)	14 (4.1)
Lifetime number of sexual partners	
<10	245 (78.3)
≥10	68 (21.7)
Lifetime number of oral sexual partners	
<3	340 (98.3)
≥3	6 (1.7)
Severe periodontitis	67 (19.4)
Time of last tooth brushing (before sample collection)	
0–4 h	137 (39.6)
≥5 h	209 (60.4)
Type of toothbrush	
Soft or super soft	112 (34.5)
Medium	186 (57.2)
Hard	27 (8.3)
Use of mouthwash on the day of the interview	157 (45.6)

### 3.2 Prevalence of oral HPV and method of assessment

The overall HPV prevalence of oral HPV infection in the study population was 4.3% (*n* = 15) using the oral rinse detection method and 5.8% (*n* = 20) using cytobrush. The prevalence of high‐ and low‐risk types was also lower for oral rinse (0.6% and 2.6%, respectively) than for cytobrush (0.9% and 3.5%, respectively [data not shown]). The sensitivity and specificity of cytobrush to detect oral HPV were 65% (95% CI: 41.3%–89.0%) and 97% (95% CI: 95.0%–98.8%), respectively, using oral rinse as the gold standard collection method. According to the area under the ROC curve, the diagnostic performance for cytobrush was 0.83. The *κ* statistic between collection methods was 0.61 (95% CI = 0.71–0.94).

Some differences were observed in the agreement between collection methods among persons positive to any consensus probes. Only 9 of these 25 individuals (36%) had the same results with oral rinse and cytobrush (Table 2). Compared with cytobrush, the oral rinse detection method classified more participants as HPV negative (40% vs. 20%) but fewer adults with low‐risk HPV (40% vs. 52%) and high‐risk HPV (12% vs. 16%) types. The cytobrush detected only one participant as having high‐risk HPV (HPV 33), whereas the oral rinse method detected two adults as having high‐risk HPV (HPV 16 and HPV 33). The low‐risk HPV types detected using oral rinse were HPV 32/42, 70, 71, 72, 81, 82, 84, and Mix 1. The same types were detected using cytobrush except for HPV 72 (Table 2).

**Table 2:** HPV types detected among oral HPV‐positive cases by collection method (*n* = 25)

	HPV type detected, by collection method	
Positive cases	Oral rinse	Cytobrush
At least one high‐risk type		
1	16	Negative
2	33, 81	33, 81
At least one low‐risk type		
3	32/42	32/42
4	33	Unknown^a^
5	Negative	58
6	Negative	32/42
7	32/42	32/42
8	32/42	32/42
9	Negative	39
10	Mix 1^b^	Mix 1
11	72	Negative
12	Negative	72
13	71	71
14	Negative	82+, 84
15	32/42	32/42
16	70	70
17	Negative	58
18	Unknown	61
19	70	70
Only unknown types		
20	Unknown	Negative
21	Unknown	Negative
22	Unknown	Negative
23	Negative	Unknown
24	Negative	Unknown
25	Negative	Unknown

Abbreviation: HPV, human papilloma virus.

^a^ Positive to HPV type of unknown significance; results positive for the consensus probes but negative for the specific HPV type probes.

^b^ Mix 1 includes HPV types 7/13/40/43/44/55/74/91.

### 3.3 Association of oral HPV infection and periodontitis

When using the oral rinse, participants with severe periodontitis had significantly higher adjusted odds of oral HPV infection (OR = 3.23, 95% CI = 1.06–9.84) than those without periodontitis after controlling for age, gender, time since last tooth brushing, and number of sexual partners. Based on cytobrush, individuals with severe periodontitis also had a higher, but statistically nonsignificant odds of oral HPV infection (OR = 1.96, 95% CI = 0.68–5.65, Table 3).

**Table 3:** Logistic regression models of the association between periodontitis and oral HPV infection by collection method (*n* = 346)

	HPV status			
Detection method	Positive, *n* (%)	Negative, *n* (%)	Crude OR (95% CI)	Adjusted OR (95% CI)^a^
Oral rinse				
Severe periodontitis	7 (10.5)	60 (89.5)	3.50 (1.25–9.77)	3.23 (1.06–9.84)
Non‐ severe periodontitis	9 (3.2)	270 (96.8)	1.00	1.00
Cytobrush				
Severe periodontitis	6 (9.0)	61 (91.0)	2.01 (0.74–5.51)	1.96 (0.68–5.65)
Non‐ severe periodontitis	13 (4.7)	266 (95.3)	1.00	1.00

Abbreviations: CI, confidence interval; HPV, human papilloma virus; OR, odds ratio.

^a^ Adjusted by age, gender, time since last tooth brushing, and number of sexual partners.

## 4 DISCUSSION

The prevalence of HPV infection was slightly lower with the oral rinse method (4.3%) than the cytobrush (5.8%); the cytobrush detected a higher proportion of low‐risk and high‐risk infections among HPV positive individuals. Whether the prevalence of HPV types in the gums is somewhat higher than in the oral cavity remains unknown. Our study also showed fair to good agreement between cytobrush and oral rinse as collection methods for detecting oral HPV (*κ*: 0.60, 95% CI = 0.71–0.94). Contrary to our findings, a study among men who have sex with men participating in a longitudinal study for oral HPV infection in Italy found a significantly lower rate of positivity for any HPV infection in oral brushing than oral rinse. Moreover, the Italian study found a lower agreement between rinse and oropharyngeal brushing for HPV detection (*κ* = 0.14, 95% CI: 0.07–0.21). The differences between these studies may be due to variations in cytobrush collection methodology; our samples were self‐collected, while samples in the Italian study were collected by otolaryngologists. The participants in our study gently brushed the gums' inner and outer areas for at least 10 strokes. In contrast, participants in the Italian study used only two cytobrushes—one to collect a sample from the oral cavity (i.e., hard palate, gums, front two‐thirds of the tongue, and floor of the mouth below the tongue) and the second from the oropharynx (i.e., soft palate, base of the tongue, and tonsils or tonsillar region). However, it is unclear how many strokes were done with each cytobrush. Finally, the Italian study was performed on a smaller sample of men who have sex with men, whereas we selected men and women from the general population of Puerto Rico. Low overlapping of HPV detection observed in our study between the oral rise and the cytobrush may be indicative of the different anatomical areas sampled. The oral gargle method collected cells from both the mouth and oropharynx, while the cytobrush specifically targeted the gums.

Despite the present study showed that the strength of the association between HPV infection and severe periodontitis varied slightly according to the collection method, the direction was the same. A stronger association with severe periodontitis was observed with oral rinse samples (OR = 3.23, 95% CI = 1.06–9.84) than with cytobrush collected samples (OR = 1.96, 95% CI = 0.68–5.65). The lower prevalence of HPV could influence the lack of statistical significance with cytobrush detected through this method, which could affect the power of our study to detect this association. These findings are consistent with previous findings in Puerto Rico and studies in other populations (Ortiz et al., 2018, 2019). For example, we showed a positive association between severe periodontitis and oral HPV infection in Puerto Rico (OR = 2.9, 95% CI: 1.0–8.4) based on the entire sample of the parent study (Ortiz et al., 2018). Although a study based on the 2009–2010 and the 2011–2012 NHANES data did not find an association between periodontitis and oral HPV infection (OR = 1.04, 95% CI: 0.63–1.73), periodontitis severity was not assessed (Wiener et al., 2015). Both of these studies collected oral HPV specimens using oral rinse samples following the NHANES methodology. To our knowledge, no studies have evaluated this association using cytobrush.

Our study had some strengths and limitations. Periodontitis and oral HPV testing were assessed using a validated methodology to help us increase the study's internal validity. In addition, the sample size was larger than in most previous reports. However, given the low prevalence of oral HPV infection in the study population, we could not stratify the participants by specific HPV types. Since the parent study used a convenience sample, the generalizability of the results is limited. The HPV samples were self‐collected, which could have introduced variability in the sample collection between participants, particularly for cytobrush samples were some studies have used trained physicians for sample collection (Fuster‐Rossello et al., 2014). However, these differences are expected to be minimal since self‐collected samples were obtained in the presence of a trained study coordinator, and complete image instruction was provided to every participant before getting the samples.

## 5 CONCLUSION

In conclusion, there was a fair to good agreement between the two collection methods for oral HPV detection. Although a positive association between HPV and periodontitis severity was observed, the association's strength and statistical significance varied according to the collection method. Additional research in high‐risk populations is needed to determine which detection method is most suitable and accurate for detecting oral HPV infection, as differences in study results could be influenced by the study methodology.

## CONFLICT OF INTERESTS

The authors declare that there are no conflict of interests.

## AUTHOR CONTRIBUTIONS

Conceptualization, supervision, funding acquisition, and project administration was performed by Ana P. Ortiz and Cynthia M. Pérez. Study design and data acquisition was performed by José Vivaldi‐Oliver, Elba C. Díaz‐Toro and Maira A. Castañeda‐Avila. Study design, data analysis and interpretation was performed by Maira A. Castañeda‐Avila, Ana P. Ortiz, and Cynthia M. Pérez. Writing original draft of the manuscript was performed by Maira A. Castañeda‐Avila. All the authors read and approved the final manuscript.

## Data Availability

The data are not publicly available due to privacy or ethical restrictions.
